# Sigmoid colon perforation in the patient with granulomatosis with polyangiitis

**DOI:** 10.1186/s40792-019-0646-1

**Published:** 2019-05-30

**Authors:** Jun Iwabu, Tsutomu Namikawa, Hiroyuki Kitagawa, Toshichika Kanagawa, Junko Nakashima, Kazuhiro Hanazaki

**Affiliations:** 1 Department of Surgery, Kochi Medical School, Nankoku, Kochi 783-8505 Japan; 2 Laboratory of Diagnostic Pathology, Kochi Medical School Hospital, Nankoku, Kochi Japan

**Keywords:** Granulomatosis with polyangiitis, Sigmoid colon perforation, Gastrointestinal involvement

## Abstract

**Background:**

Granulomatosis with polyangiitis (GPA) induces respiratory tract and kidney granulomatous inflammation due to small-vessel vasculitis. However, gastrointestinal involvement, and especially colon perforation, is rare.

**Case presentation:**

A 40-year-old man diagnosed with GPA was admitted to our hospital for GPA management. He was started on anti-cluster of differentiation 20 antibody (rituximab) therapy after admission and suffered severe abdominal pain 2 weeks later. A clinical diagnosis of sigmoid colon perforation was made, and we performed sigmoid colon resection with colostomy. Histopathological examination revealed loss of vascular wall and neutrophil infiltration. He was discharged from the hospital after intravenous immune globulin therapy.

**Conclusions:**

Although gastrointestinal involvement is rare in GPA, severe complications require surgical intervention. Bowel perforation should be considered an important complication of GPA.

## Background

Granulomatosis with polyangiitis (GPA, formerly known as Wegener’s granulomatosis) is a systemic anti-neutrophil cytoplasmic antibodies-associated vasculitis that may result in life-threatening organopathies such as nephritis and pulmonary infiltration [1]. However, although there have been reports of colon perforation in GPA, gastrointestinal involvement is rare [2]. Here, we present a case of sigmoid colon perforation with GPA.

## Case presentation

A 40-year-old Japanese man was referred to Kochi Medical School Hospital for treatment of GPA. The diagnosis of GPA had been made by symptoms of multiple lung nodule, otitis media, sinusitis, skin ulcer, and periocular granuloma 20 years ago. Furthermore, histopathological finding from skin ulcer revealed granulomatous vasculitis and blood examination showed positive of cytoplasmic anti-neutrophil cytoplasmic antibody (C-ANCA). He had been treated for GPA by his general practitioner with corticosteroids and methotrexate for 20 years. He was started on anti-cluster of differentiation (CD) 20 antibody (rituximab) and prednisolone combination therapy due to fever and advance of skin vasculitis, after admission to our hospital. Rituximab was injected day 1 (500 mg/body), and prednisolone was taken orally from day 1 to 14 (30 mg/body). Skin vasculitis had improved slightly; however, he suffered high-grade fever and severe abdominal pain at day 14. Blood analysis revealed high levels of creatinine (1.93 mg/dL; normal range, 0.65–1.07 mg/dL) and blood urea nitrogen (24 mg/dL; normal range, 8–20 mg/dL). He was also found to have leukocytosis (white blood cell count, 30,200/μL; normal range, 3,300–8,600/μL), a C-reactive protein level of 11.41 mg/dL (normal range, < 0.14 mg/dL), and a procalcitonin level of 0.72 ng/mL (normal range, < 0.05 ng/mL).

**Fig. 1:**
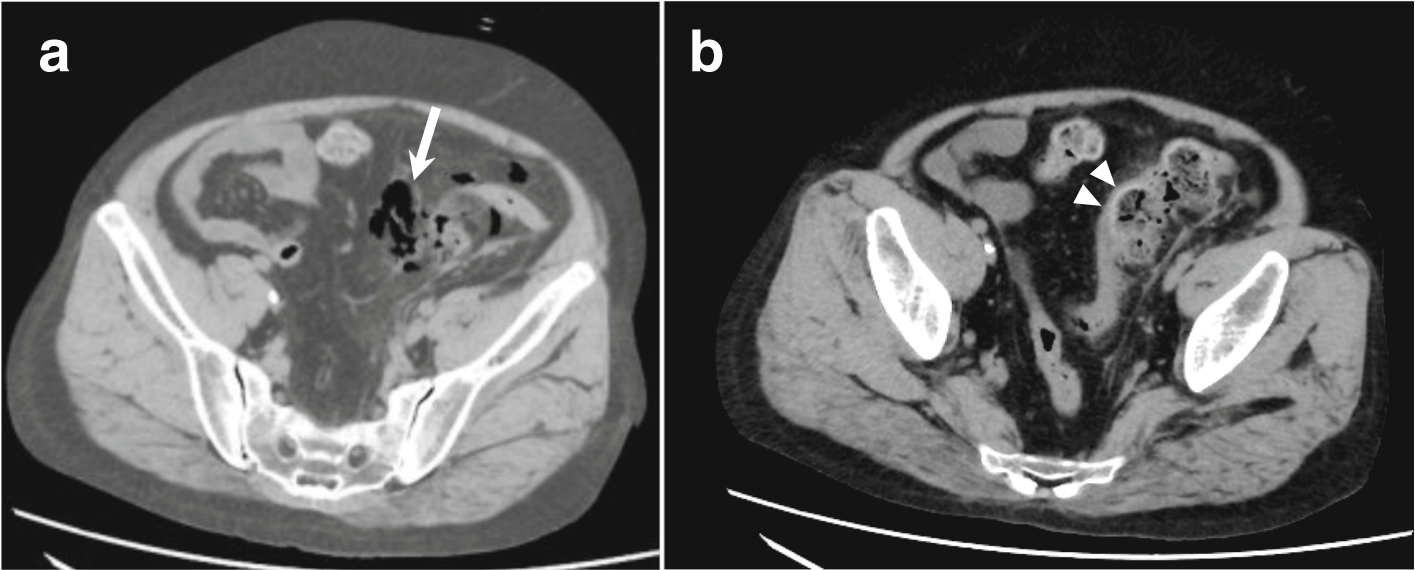
Abdominal contrast-enhanced computed tomography showing a colonic perforation. Mesenteric emphysema (**a**, arrow) and inflammation (**b**, arrowhead) of the sigmoid colon are visible

**Fig. 2:**
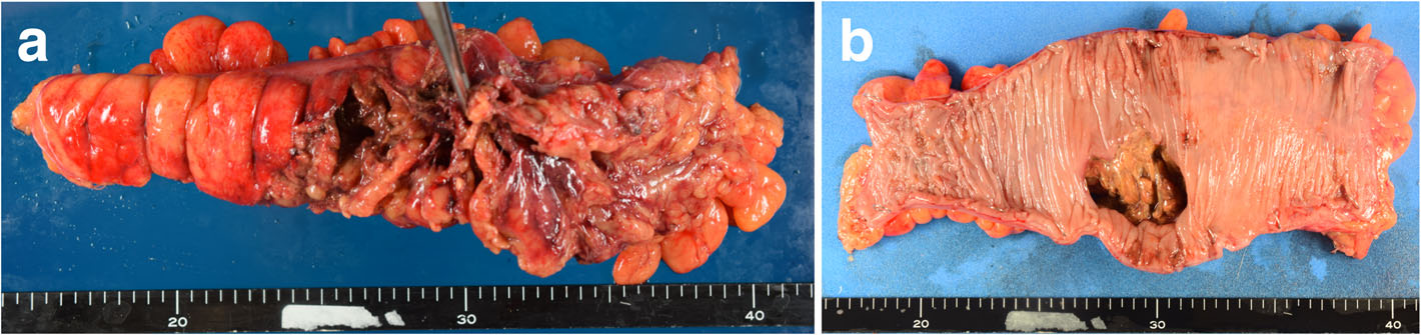
Macroscopic appearance of the resected specimen. The surface of the serosal side showed edematous due to inflammation (**a**). The surface of the mucosal side shows a perforation of 4 cm in diameter. The mucosae around the perforation site are intact without ischemic change (**b**)

**Fig. 3:**
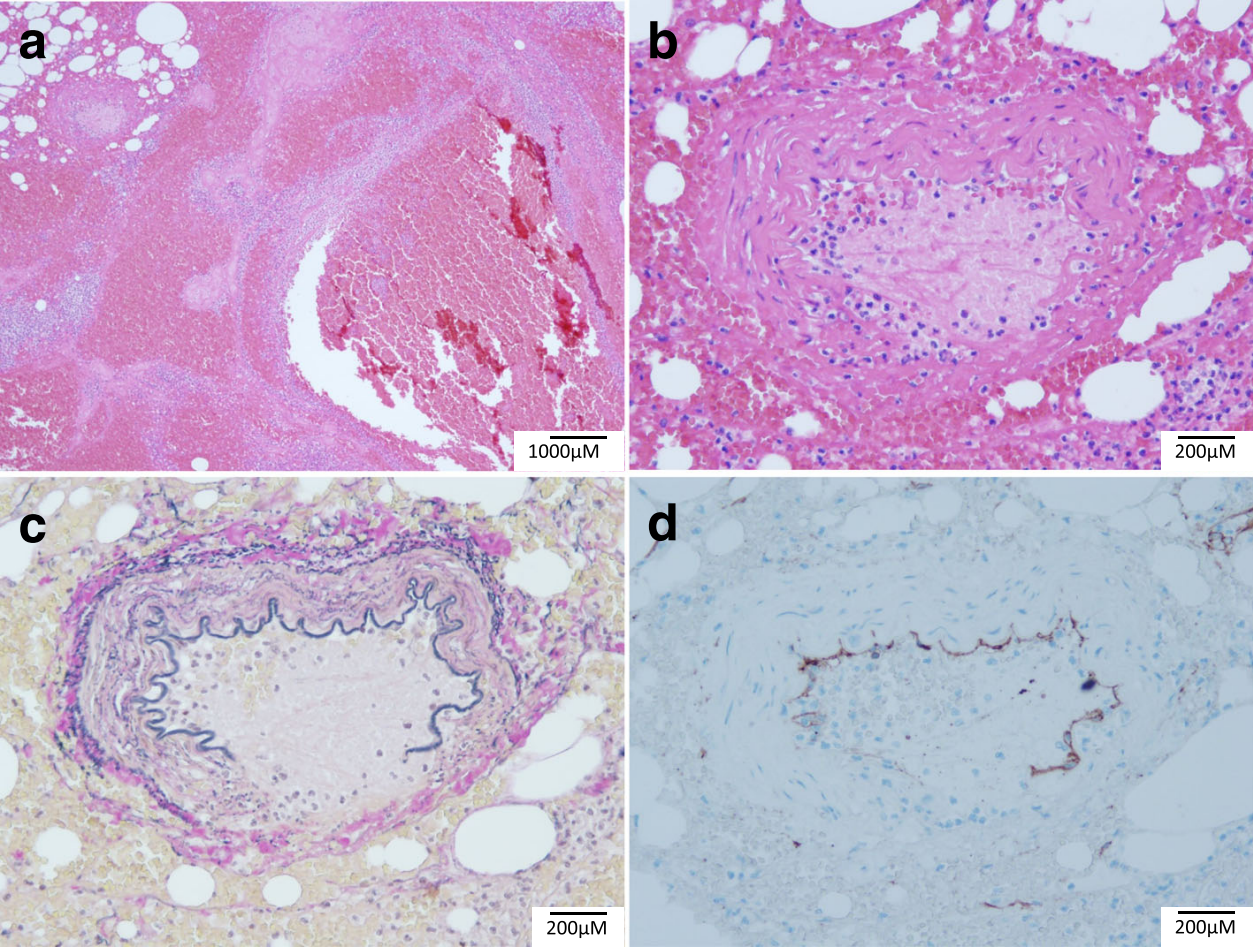
Pathological findings of the resected specimen. Histological sample of the colon shows ulcerative lesions and fibrinoid necrosis (**a**, hematoxylin and eosin [HE] staining, × 40). Fibrinoid necrosis and neutrophil infiltration of the small vessel (**b**, HE staining, × 200) and loss of vascular wall (**c**, Elastica van Gieson staining, × 200; **d**, cluster of differentiation 34 staining, × 200) are demonstrated

Computed tomography showed mesenteric emphysema of the sigmoid colon with inflammatory changes in the surrounding tissue (Fig. 1). After a clinical diagnosis of sigmoid colon perforation was made, we performed emergency surgery. During surgery, a sigmoid colon perforation of 3 cm in diameter was found. Although descending and sigmoid colon was edematous due to inflammation, there were no abnormal findings in the other part of the colon and small intestine. Resection of 23 cm of sigmoid colon and rectum with colostomy formation was performed in accordance with Hartmann’s approach (Fig. 2). Histological examination of the colon showed ulcerative lesions without cancer (Fig. 3a), and vessels around the ulcer showed loss of wall structures and neutrophil infiltration (Fig. 3b–d).

The patient had a postoperative complication of superficial surgical site infection, which was treated by opening, draining, and debriding the wound. After the wound healed, he was treated with intravenous immune globulin. He was discharged from our hospital 55 days after the emergency surgery with no other complications. The patient was asymptomatic and received medical treatment for GPA after the sixth postoperative month.

## Discussion

GPA is characterized by necrotizing vasculitis and granulomatous inflammation. The disease is diagnosed based on findings of nasal or oral inflammation, nodules on chest radiographs, abnormal urinary sediment, and granulomatous inflammation on biopsy of the artery or perivascular area [3]. These findings are indicative of life-threatening organopathies, such as nephritis and pulmonary infiltration. However, gastrointestinal involvement of GPA is rare, with a reported incidence rate of 5–11% [4].

There is published literature on gastrointestinal involvement in systemic necrotizing vasculitis. Earlier reports showed that GPA patients presented with diarrhea, hematochezia, and gastrointestinal ulcers [5–7]. Pagnoux et al. reported the outcome of gastrointestinal involvement in 62 patients with systemic necrotizing vasculitis which include GPA, polyarteritis nodosa, Churg-Strauss syndrome, microscopic polyangiitis, and rheumatoid arthritis-associated vasculitis [5]. In their report, 9 of 62 patients (15%) had bowel perforation; however, there was no patient with GPA in 9 patients and the longest interval from diagnosis in their cohort was only 68 months. Furthermore, less than 20 cases of bowel perforation associated with GPA were reported in published literature in English [8–21]. Table 1 shows the clinicopathological features of the 15 reported cases [8–21] as well as those of the present case. The median patient age was 44 years (range, 19–69 years), and the male-to-female ratio was 11:4. The reported perforation sites were the jejunum in 2 cases, the ileum in 10 patients, and the colon in 4 patients. Histological analysis of the lesions revealed 9 and 4 instances of vasculitis and ulceration, respectively. To the best of our knowledge, ours is the second case of isolated colon perforation. There were no reports about colon perforation with GPA patients after rituximab administration without our patient. Furthermore, previous reports indicate that gastrointestinal symptoms tend to occur within 5 years of the initial diagnosis. In our patient, sigmoid colon perforation occurred 20 years after the initial diagnosis. This case may be categorized as another specific colon perforation group with GPA in terms of long treatment period and rituximab administration.

**Table 1:** Reported cases of intestinal perforation associated with granulomatosis with polyangiitis

Reference	Age/sex	Perforation site	Disease duration (months)	Drug	Pathology	Prognosis
Our case	40/M	Colon	240	P + M + R	Vasculitis	Survival
Toh et al. [8]	19/F	Small bowel	1	P + R	Vasculitis	Survival
Akbulut [9]	47/M	Ileum	18	P + C	NS	Death
Samim et al. [10]	35/M	Jejunum	4	P + C	Ulceration	Survival
Yildirim et al. [11]	32/M	Ileum	0.5	P + C	Vasculitis	Death
Deniz et al. [12]	44/M	Ileum	1	NS	Vasculitis	Survival
Shaikh et al. [13]	44/F	Ileum, colon	2	P	Vasculitis	Survival
Strivens et al. [14]	54/F	Ileum, colon	1.5	P + C	Vasculitis	Survival
Akca et al. [15]	56/M	Ileum	3	P + C	Ulceration	Survival
Skaife at al. [16]	69/M	Jejunum	0.2	P + C	NS	Death
Srinivasan and Coughlan [17]	56/F	Ileum	1	P + C	Vasculitis	Survival
Storesund et al. [18]	26/M	Colon	18	P + C	Vasculitis	Survival
Tokuda et al. [19]	37/M	Ileum	24	P + C	Vasculitis	Survival
Geraghty et al. [20]	46/M	Ileum, colon	1	P + C	Ulceration	Death
McNabb et al. [21]	50/M	Ileum	9	P + A	Ulceration	Survival

*P* prednisolone, *M* methotrexate, *R* rituximab, *C* cyclophosphamide, *A* azathioprine, *NS* not stated

A plausible reason for the perforation is the advanced nature of the polyangiitis. We investigated the histopathological findings that were not contradictory to the diagnosis of GPA using Elastica van Gieson and CD34 staining protocols. The prognosis of GPA has improved with effective induction therapy using corticosteroids combined with cyclophosphamide or rituximab, with 5-year survival rates > 80% [22]. Therefore, careful examination at follow-up with emphasis on assessing possible gastrointestinal involvement is necessary for GPA patients over the long term.

Another likely reason for the perforation is the effect of rituximab. There are no reports of bowel perforation following rituximab administration. However, there are reports of bowel perforation associated with rituximab in post-transplant lymphoproliferative disorder [23, 24]. In 2006, Roche Pharmaceuticals issued a warning regarding the risk of intestinal perforation following rituximab administration.

Compared to previous reported cases, the interval from GPA diagnosis to colon perforation was exceptionally long. Former colon perforation cases with GPA patients might be involved in granulomatosis strongly. In our patient, long-term corticosteroids and methotrexate administration might have contributed to the weakness and thinning of the intestinal wall. The colon perforation was likely to be occurred by combination of intestinal wall weakness, GPA progression, and rituximab administration.

To avoid severe gastrointestinal complications in GPA patients, it is crucial that severe abdominal symptoms are not missed. Especially when GPA progression or rituximab administration starts, it is important to beware of symptoms such as high grade and severe abdominal pain. Gastrointestinal perforation should be regarded as an important complication of GPA.

## Conclusions

GPA with sigmoid colon perforation is rare, but early diagnosis and rapid treatment are necessary when it occurs.

## Abbreviations

CD: Cluster of differentiation
GPA: Granulomatosis with polyangiitis
